# New insights into fibrosis from the ECM degradation perspective: the macrophage-MMP-ECM interaction

**DOI:** 10.1186/s13578-022-00856-w

**Published:** 2022-07-27

**Authors:** Xiangyu Zhao, Jiayin Chen, Hongxiang Sun, Yao Zhang, Duowu Zou

**Affiliations:** 1 Department of Gastroenterology, Ruijin Hospital, Shanghai Jiao Tong University School of Medicine, Shanghai, China; 2 Department of Immunology and Microbiology, Shanghai Institute of Immunology, Shanghai Jiao Tong University School of Medicine, Shanghai, China; 3 The State Key Laboratory of Oncogenes and Related Genes, Shanghai Jiao Tong University School of Medicine, Shanghai, China

**Keywords:** Fibrosis, Extracellular matrix, Macrophage, Matrix metalloprotease, Matricryptin

## Abstract

Fibrosis is a pathological feature of a variety of chronic inflammatory diseases that can affect almost all organs, which can cause severe consequences and even lead to death. Fibrosis is characterized by the excessive accumulation of extracellular matrix (ECM) due to disruption of the balance between ECM production and degradation. Although overabundance of ECM proteins has long been the focus of studies on fibrosis, another facet of the problem—impaired degradation of the ECM—is gaining increasing attention. Matrix metalloproteinase (MMP) and the tissue inhibitor of metalloproteinase (TIMP) system is the main molecular system contributing to ECM degradation, and macrophages are the major regulators of ECM. However, the relationship among macrophages, the MMP/TIMP system and the ECM is not fully understood in the context of fibrosis. Here, we discuss in detail the role played by the ECM in the development of fibrosis and highlight the macrophage-MMP-ECM interaction that is involved in fibrogenesis and may be a potential therapeutic target for fibrosis.

## Introduction

Fibrosis results from abnormal wound healing under chronic inflammation or during ongoing injury and is characterized by the excessive accumulation of extracellular matrix (ECM) in and around inflamed or damaged tissue, which may impair the physiological function of the affected tissue and eventually lead to organ failure. [1, 2] Fibrosis can affect multiple organs, such as the liver, kidney, heart, lung, skin and intestine, and can be triggered by a variety of diseases, including cirrhosis, chronic kidney disease, heart failure, idiopathic pulmonary fibrosis (IPF), scleroderma and inflammatory bowel disease (IBD). [3] The annual incidence of major fibrotic diseases is approaching 5% worldwide and accounts for more than one-third of the annual mortality in industrialized countries [4, 5].

The ECM plays an important role in the pathogenesis of fibrosis. The dynamic equilibrium between ECM production and degradation plays an important role in the maintenance of tissue homeostasis [6]. However, in fibrotic diseases, in addition to the overabundance of ECM proteins, inhibition of the degradation pathway is crucial to the development of fibrosis [7]. Under physiological conditions, proteases, such as matrix metalloproteinases (MMPs), contribute to the maintenance of this equilibrium by hydrolyzing ECM components, such as collagen, into small peptides. The dysfunction of proteases impairs the degradation of ECM components in fibrotic diseases, resulting in excessive ECM deposition in fibrotic tissues, which becomes a direct factor leading to fibrosis [8].

Macrophages, the main producers of proteases in tissues, can regulate both the progression and regression of fibrosis. Macrophages release cytokines to regulate the immune response; phagocytize pathogens, apoptotic cells and ECM; and secrete proteases and their inhibitors to mediate tissue remodeling [9]. Moreover, macrophages can interact with other cells involved in fibrosis, such as activating other immune cells through antigen presentation and inducing fibroblast proliferation or apoptosis, thus regulating the processes of inflammation and fibrosis [10].

As noted above, the interaction of ECM, proteases and macrophages plays a core role in the pathogenesis of fibrosis. However, the interaction pathway and regulatory mechanism of the macrophage-MMP-ECM interaction are unclear. Therefore, we first summarize the mechanism of multiple ECM components in fibrogenesis. Then, we describe a certain kind of macrophage that is critical for ECM degradation in fibrosis. Finally, we elucidated the interactions among macrophages, MMPs and the ECM, which may provide clues for the treatment of fibrosis.

### ECM and fibrosis

The ECM is a three-dimensional, noncellular structure composed of approximately 300 proteins, including 43 collagen subunits, 36 proteoglycans and nearly 200 complicated glycoproteins [11]. Depending on its location, composition, and function, the ECM is divided into two main types: the interstitial connective tissue matrix and the basement membrane [12]. The interstitial connective tissue matrix surrounds cells and is mainly composed of fibrillar proteins (including fibrillar collagen, fibronectin, elastin and vitronectin), glycoproteins (fibrillins, tenascins, etc.), matricellular proteins (e.g., CCN (cyr61, ctgf, nov) proteins and thrombospondins) and polysaccharides, such as hyaluronan. The main function of the interstitial connective tissue matrix is formation of a meshwork that connects structural cell types within tissues (which is mainly realized through fibrillar proteins). Moreover, alteration to the structure of the interstitial connective tissue matrix (comprising, for example, fibronectin and heparin) can regulate cell–cell or cell–matrix interactions by releasing growth factors (e.g., active or latent TGF-β and BMP-2) that had been originally bound within the matrix [6, 13]. In contrast, the basement membrane is composed mainly of collagen IV, laminins, heparan sulfate proteoglycans, etc. Its main physiological functions are to separate epithelial cells from the surrounding matrix and to regulate cell differentiation by interacting with cell surface receptors [14]. Both basement membranes and interstitial matrices create tissue-specific niches that affect the stemness and differentiation of progenitor cell populations, as well as the proper functions of tissue-specific differentiated cell types [12].

ECM (mainly fibronectin, collagen and proteoglycan) and its dynamic changes in composition and structure are important to the pathogenesis and development of fibrosis. Excessive deposition of ECM at a fibrotic site is a common pathological feature, and the identification of this deposition is the gold standard for the diagnosis of fibrosis. Generally, in solid organs (liver and kidney fibrosis), elastin deposition in vessel walls leads to local blood supply disorders and damage to parenchymal cells (e.g., hepatocytes and glomerular and tubular epithelial cells), which eventually result in organ dysfunction [15, 16]. In contrast, fibrosis in cavernous organs may lead to thickening and stiffening of the wall. For instance, in cardiac fibrosis, large amounts of ECM (mainly various isoforms of collagen) are deposited mainly in the ventricular wall and reduce wall compliance, ultimately leading to a decrease in the ejection capacity of the heart [17, 18]. Specifically, different ECM components play unique roles in each type of fibrotic disease. Type I and III collagen and extra-domain A fibronectin (EDA-FN fibronectin) form the pathological core in IPF, which continuously activates fibroblasts in lesions and ultimately leads to fibrosis. In cardiac fibrosis, tenascin-C activates cardiac fibroblast TLR4 signaling mediated by damage associated molecular patterns (DAMPs) signaling, thereby promoting inflammatory and profibrotic responses, while perlecan promotes fibroblast migration and survival by activating α2β1 signaling [19, 20].

ECM is not only a pathological feature of fibrosis but is also an exacerbating factor of fibrosis by either affecting the course of primary disease or by facilitating ECM accumulation. First, when accumulated, multiple components of the ECM may worsen primary fibrotic disease. Reese-Petersen AL’s team found that type I and type III collagen expression was upregulated in the left atrium of patients with atrial fibrillation (AF), and the accumulated collagen affected the electrical conductance of the entire heart by influencing atrial myocyte action potential timing and conductivity, ultimately resulting in more frequent and worsening AF [21]. Second, deposition of ECM creates a positive feedback loop in fibrotic diseases. In the case of idiopathic pulmonary fibrosis, a fibrosis progression mechanism involves increased matrix stiffness via the deposition of ECM, which leads to excessive ECM abundance in the tissue microenvironment. Specifically, as tissue fibrosis proceeds, lysyl oxidase catalyzes the formation of aldehydes from lysine residues in collagen and elastin, and then, these residues react with each other to form cross-links between collagens, which subsequently increases matrix stiffness and inhibits ECM degradation. [22] Moreover, enhanced mechanical stress can act as a signal to induce more ECM production through the activation of downstream pathways (e.g., TGF-β1 and Wnt-β-catenin) and the epithelial-mesenchymal transition (EMT) [23]. Even worse, ECM can maintain the activation of ECM-secreting cells (mainly myofibroblasts) through the downregulation of miR-29, eventually forming a vicious cycle and accelerating fibrosis [24]. Numerous studies have identified miR-29 as a master fibro-miRNA and negative regulator that plays crucial roles in fibrotic diseases such as cardiac fibrosis, renal fibrosis and pulmonary fibrosis. Researchers have indicated that miR-29 members target at least 16 ECM-related genes, such as TGF-β, MAPK and Notch. miR-29b and miR-29c block TGF-β signaling by base pairing with the coding sequence (CDS) in TGF-β exon 3 to prevent cardiac fibrosis [25].

In chronic inflammation, the production and degradation of ECM deviate from the equilibrium, eventually leading to irreversible excess deposition of ECM in tissues and organs. [26] Although hyperactivation of ECM production has been widely studied [27], an increasing number of studies have shown that targeting ECM degradation is a potential strategy for the treatment of fibrosis. Products of ECM degradation can be used as biomarkers of fibrotic diseases and to help in diagnosing and differentiating diseases. In a randomized controlled trial (RCT) including patients with ulcerative colitis (UC) and Crohn’s disease (CD), significant differences in the concentrations of degraded collagen I and III-IV (C1M, C3M, and C4M) and MMP-degraded vimentin (VICM) in these patients enabled the accurate differentiation of CD, UC and non-IBD patients. [28] A number of in vivo experiments and clinical cohort studies have targeted ECM degradation for the treatment of fibrosis. In a mouse model of bleomycin-induced pulmonary fibrosis, the collagen component of pulmonary fibrosis was found to be effectively degraded under relaxin induction, and then, the severity of pulmonary fibrosis was decreased [29]. In a clinical RCT study in IPF patients, IW001, a drug targeting anti-collagen V (col(V)) antibodies, was tested and found that the cohort receiving the highest dose showed better viability, suggesting that IW001 may be a new therapeutic approach for col(V)-reactive IPF patients [30]. These studies suggest that targeting ECM degradation to alleviate fibrosis is a promising therapeutic strategy, more research is needed in the future.

### Fibrolytic macrophages in fibrosis

Macrophages are the drivers of fibrosis in most organs such as liver, lung and skin , but in fact, the function of macrophages in injury repair and fibrosis is plastic and complicated [31]. They can be stimulated by different signals in the microenvironment and perform corresponding functions [29]. Macrophages exhibit two different polarization phenotypes in vitro. Treatment with interferon-γ and lipopolysaccharides generated classically activated macrophages (the M1 type), while treatment with interleukin (IL)-4/IL-13 polarized macrophages into alternatively activated macrophages (the M2 type) [32]. In general, the M1 type is considered to be proinflammatory, while the M2 type is considered to be anti-inflammatory [33].

However, M1/M2 status is, in fact, an oversimplified classification that does not adequately describe the complex role played by macrophages in the fibrosis of different organs and at different stages. Therefore, an increasing number of studies have classified macrophages according to their specific functions in tissue injury and fibrosis. In the early stage of tissue injury, a subset of macrophages can promote fibrosis by releasing proinflammatory cytokines, inducing the activation and proliferation of ECM-producing cells and recruiting more macrophages to an injury site; hence, these cells are called profibrotic macrophages [34]. In addition, Haider et al. found that macrophages may contribute to postinfarct cardiac fibrosis through conversion into collagen-producing fibroblast-like cells, which may be promising targets to modulate fibrotic responses after myocardial infarction [35]. During the resolution phase, another subset of macrophages can prevent or reverse fibrosis by degrading ECM components, inhibiting the activity of ECM-producing cells and clearing apoptotic cells. These macrophages subset are called anti-fibrotic macrophages [36]. The continuous activation of profibrotic macrophages and inhibition of anti-fibrotic macrophages disrupts the balance between pro- and anti-fibrotic macrophages, which may be an important mechanism by which the repair process deviates from the normal progression, leading to excessive ECM deposition and eventually to fibrosis [37].

In this article, we describe macrophages from distinct organs that serve similar functions to express ECM degradation-related genes to promote the resolution of fibrosis as fibrolytic macrophages, and their characteristics are described in Table 1. [38] (Table 1) Fibrolytic macrophages express a variety of proteins involved in ECM degradation, including secretory proteases, phagocytic receptors and enzymes critical for the intracellular digestion of phagocytosed ECM fragments [39]. Fibrolytic macrophages can express several proteolytic enzymes. In addition to a series of MMPs, fibrolytic macrophages can secrete cathepsins to degrade collagen in the ECM in pulmonary fibrosis [40]. Fibrolytic macrophages also express receptors or soluble proteins involved in ECM protein uptake, including integrin, MFGE8, MRC1, etc. [41, 42]. Fibrolytic macrophages are post-phagocytic, and they are larger in size and contain ingested apoptotic debris. [43] Moreover, fibrolytic macrophages downregulate gene clusters involved in collagen organization and focal adhesion in peritoneal fibrosis. [44] Conclusively, macrophage subsets with similar ECM-degrading functions have been found in many fibrotic organs, but their specific features, such as surface markers, phenotypes, gene expression, functional pathways and regulatory mechanisms in different tissue microenvironments are still unclear. Therefore, further studies are needed to identify the characteristics of fibrolytic macrophages, which may facilitate in-depth investigation into the mechanism of fibrosis and provide cues for anti-fibrosis treatment.

**Table 1:** Characteristics of Fibrolytic Macrophages

	Category	Related Proteins	Functions	Conditions	Reference
Anti-fibrosis↑	Fibrolysis	MMP-1, MMP-2	Degrading collagen	Atherosclerotic plaques	[45]
		MMP-14, cathepsin K		Pulmonary fibrosis	[46]
		MMP-2, MMP-9		Renal fibrosis	[47]
		MMP-13		Liver fibrosis	[48]
	ECM uptake	Integrin α2β1	Involved in soluble collagen degradation and phagosome maturation mediated by uPARAP/Endo180 or mannose receptor	In vitro	[49]
		Mannose receptor	Binding and internalizing collagen IV and gelatin in a carbohydrate-independent manner	In vitro	[50]
		uPARAP (Endo180)	Binding collagen through its Endo180 FNII domain	In vitro	[51]
		Mfge8	Binding collagen through its discoidin domains; targeting collagen uptake and digestion by macrophages	Pulmonary fibrosis	[41]
		Stabilin-1	A homeostatic scavenger receptor binding ECM osteonectin	Liver fibrosis	[42]
	Proresolution cytokine	IL-10	Preventing macrophage production of proinflammatory cytokines; preventing the development of Th1-type and Th2 T-cell responses; promoting regulatory T-cell population	Renal fibrosis	[47]
		HGF	Enhancing hepatocyte proliferation after liver injury	Liver fibrosis	[52]
		CXCL1	Recruiting collagenase-producing neutrophiles to degrade the ECM	Liver fibrosis	[53]
Profibrosis↓	Profibrotic cytokine	TGF-β1	Regulating fibroblast proliferation, migration, activation and differentiation	Liver fibrosis	[43]
		TNF-α	Promoting hepatic stellate cell (fibroblast) survival and activation; upregulating expression of TIMP	Liver fibrosis	[54]
		CXCL2 (MIP-2)	Recruiting neutrophiles; promoting fibroblast proliferation	Pulmonary fibrosis	[55]

*uPARAP* urokinase plasminogen activator receptor-associated protein, *Mfge8* milk fat globule epidermal growth factor 8, *HGF* hepatic growth factor, *MIP-2* macrophage inflammatory protein 2

Fibrolytic macrophages consist of macrophage subsets from multiple sources, of which two main sources are the phenotypically changed tissue-resident macrophages and monocytes recruited from peripheral blood. Stimulation of upstream signaling by the tissue microenvironment can trigger the functional regulation and phenotypic change of tissue-resident macrophages, resulting in their acquisition of the anti-fibrotic phenotype. For instance, IL-4-activated macrophages displayed increased expression of cathepsin S and L and reduced activity of phagosomal NADPH oxidase 2 (NOX2), which greatly enhanced the proteolytic capacity of their phagosomes [56]. Moreover, another study found that in pulmonary fibrosis, recognition and clearance of apoptotic cells by macrophages triggered the activation of peroxisome proliferator-activated receptor-γ (PPARγ, a transcription factor essential to the alternative activation of macrophages), leading to the upregulation of proresolution cytokines such as IL-10 and hepatic growth factor (HGF), as well as the downregulation of proinflammatory cytokines (TNF-α and macrophage inflammatory protein 2) and fibrosis markers (collagen 1α2, fibronectin, α-smooth muscle actin, etc.), thus hastening the resolution of fibrosis [57]. Recruitment and transformation of monocytes from peripheral blood create another source of fibrolytic macrophages. In both murine liver and kidney fibrosis, Ly6C^hi^ (Gr1^hi^) monocytes in peripheral blood were recruited to sites of injury or inflammation [39, 54]. After phagocytosing cell debris, Ly6C^hi^ monocytes transformed into Ly6C^lo^ monocytes, which further differentiated into Ly6C^lo^ fibrolytic macrophages through the regulation of CX3CR1 expression [39]. In addition to monocyte recruitment from peripheral blood, fibrolytic macrophages also originate from macrophages recruited from adjacent areas. Deniset JF et al. showed that GATA6+ macrophages in the pericardial cavity migrated to injured heart tissues and augmented cardiac fibrosis [58].

### Macrophage-MMP-ECM interaction

The secretion of MMP and the ingestion of ECM components by macrophages are two main ECM degradation pathways in fibrosis. Therefore, we aimed to elucidate the common mechanisms and regulation of fibrolytic macrophages in fibrosis from the perspective of the interaction of macrophages, MMPs and the ECM. We also discuss how relevant mechanisms can be transformed into potential targets for anti-fibrosis therapy.

### MMPs and the ECM

MMPs are the main proteases involved in ECM degradation. Their activity is low under normal conditions but increases during repair or inflammation [59]. MMPs cleave all ECM components, including collagen, fibronectin, laminin, and gelatin, in the form of secreted or cell membrane-anchored proteins. [60] MMPs carry many functional domains: a signal peptide domain, propeptide domain, catalytic domain and hemopexin-like domain (except MMP7, MMP23 and MMP26), among which the catalytic domain exhibits a major hydrolytic function. The catalytic domain includes a highly conserved Zn2+ binding motif, in which the nucleophilic glutamate attacks the peptide bond of a substrate to hydrolyze the macromolecule [61]. The TIMP family includes four members (TIMP1-TIMP4), all of which can reversibly inhibit the activity of proteases such as MMPs. In unstimulated human peripheral blood, TIMPs are mainly expressed by monocytes, B cells and T cells [62]. The N-terminal end of TIMP folds within itself and, importantly, is wedged into the active site of MMPs, inhibiting MMP hydrolytic activity [63]. The concentration ratio of MMP and TIMP determines the total potency of protein hydrolysis, and this balance plays an important role in angiogenesis, inflammation, tissue repair and the development of fibrosis [8].

Numerous studies have demonstrated significant changes in the levels of MMP/TIMP in patients with fibrotic diseases as well as in experimental models of fibrosis. For example, patients with cardiac fibrosis exhibited significantly higher levels of free MMPs (MMP-3, MMP-9, etc.) in peripheral blood and lesions compared with healthy controls[64, 65]. In further studies, MMP- or TIMP-knockout mice were used to verify the essential role played by MMP/TIMP in the development of fibrosis. Pellicoro A’s team found that MMP-12 was secreted from the liver of mice mainly by Kupffer cells (tissue-resident macrophages in the liver). Serving as CCL4-induced liver fibrosis model, MMP-12 knockout mice showed more elastin deposition both in the early and late stages of fibrosis [66]. K J Leco et al. found that MMP activity was higher in TIMP-3-deficient mice than in wild-type mice. The altered MMP/TIMP balance resulted in enhanced collagen degradation in the peribronchiolar space and disruption of collagen fibrils in the alveolar interstitium, respectively, and both outcomes led to accelerated pulmonary fibrosis [67]. The balance of the MMP/TIMP system is critical for the homeostasis of ECM components and structure, while the disruption of this system causes dysregulation of the ECM, which further leads to the pathogenesis and development of fibrosis.

### Macrophages and MMPs

Macrophages can regulate the production and activity of MMPs. The regulation of MMP production by fibrolytic macrophages is categorized into transcriptional regulation and secretory regulation. A variety of transcription factors, such as EGR1, GATA1, and NF-κB, the AP-1 family and STAT3C are involved in the transcriptional regulation of MMPs[68]. The initial signals originated from ligands binding to integrins (INT), Toll-like receptors (TLR) and receptors for interferons (IFN), interleukin (IL)-1 (IL1R), tumor necrosis factor (TNFR), prostaglandin E2 (EP4), IL-6 (GP130) and granulocyte–macrophage colony-stimulating factor (CSFR2).Then, the phophoinositide-3 kinase (PI3K), extracellular signal-related kinases 1/2 (ERKs), p38 mitogen-activated protein kinase and c-jun N-terminal kinase (JNK) are also activated. These lead together to activation of the activator protein-1 (AP-1) and nuclear factor-κB (NF-κB) transcription factors that directly induce several MMPs and TIMP-1 [68].

The process of MMP secretion is essential to regulate MMP activity. Initially, secreted pro-MMPs are non-functional due to the interaction of the unpaired cysteine sulfhydryl group in the propeptide domain with the zinc ion-containing active site. Disruption of the bond between the sulfhydryl group of the conserved cysteine in the propeptide domain and the active site zinc ion causes activation of the latent enzyme, a mechanism called the cysteine switch of pro–MMP activation [69]. Fibrolytic macrophages play an important role in regulating this cleavage process by secreting the serine protease thrombin. The secretion of preformed MMP12 is induced when thrombin cleaves PKC-activating protease-activated receptor-1 (PAR-1) on the surface of macrophages [70].

On the other hand, MMPs can regulate the function of fibrolytic macrophages. MMPs (including MMP-1, MMP-2, MMP-3 and MMP-13) are endogenous inhibitors of macrophage recruitment in inflammation. [71] For example, MMP2 cleaves and inactivates monocyte chemoattractant protein-3 (MCP-3) in the tissue microenvironment, concurrently producing a general antagonist to chemokine receptors on macrophages, thereby inhibiting the recruitment of monocytes from peripheral blood to sites of inflammation [72]. Moreover, MMPs (including MMP-10 and MMP-28) can promote the anti-fibrotic function of macrophages [73]. In pulmonary fibrosis, MMP-28 dampens proinflammatory macrophage function while promoting M2 polarization, which augments fibrosis [54].

### Macrophages and ECM

In addition to secreting MMPs to degrade ECM, macrophages ingest ECM components and digest them through the lysosomal pathway. Integrin-mediated phagocytosis is the classical pathway involved in macrophage ECM ingestion. Furthermore, proved in renal and pulmonary fibrosis, receptor-mediated endocytosis plays an important role in ECM ingestion, and this pathway includes the urokinase plasminogen activator receptor-associated protein (uPARAP/Endo180) and CD206 pathways [74]. A recent study indicated that some extracellular soluble glycoproteins also mediate macrophage endocytosis by binding to collagen, promoting ECM clearance. In bleomycin-induced pulmonary fibrosis, milk fat globule epidermal growth factor 8 (Mfge8, a soluble glycoprotein)-knockout mice exhibited impaired collagen uptake and enhanced fibrosis severity [41].

Conversely, ECM components in the fibrotic microenvironment can regulate the function of macrophages. In fibrosis, with increases in the amount of ECM, the properties of the ECM change markedly. The accumulation of specific ECM components during fibrosis and the products of ECM degradation all make fibrotic ECM different from the ECM in normal tissue [75]. The unique properties of fibrotic ECM can affect the function of macrophages, such as their phenotype acquisition or chemotactic properties. Specific components of the fibrotic ECM can induce phenotypic changes in macrophages. Chondroitin sulfate proteoglycan (CSPG) is a component of glial scars formed after spinal cord injury. CSPG-cultured monocytes are induced to express high levels of IL-10, a sign of transformation toward the fibrolytic macrophage type [75]. Moreover, products of active ECM degradation show chemotactic or activating effects on monocytes. With the increase in ECM production during fibrosis, ECM degradation is more active, resulting in a significant increase in the number of ECM degradation products. Several proteinase families, mostly zinc metalloproteinases and serine or cysteine proteases, generate bioactive fragments during ECM remodeling. The ECM fragments produced in the process of ECM degradation (e.g., collagens, elastin, fibronectin, laminins and matricellular proteins) contain structural sites with unique biological activities that were previously inactive within ECM molecules under normal conditions [76]. These fragments are defined as matricryptins or matrikines. Although different matricryptins are derived from different sources and are of differing sizes, many matricryptins exhibit chemotactic effects on monocytes in the tissue microenvironment, including tripeptide GHK (a fragment of collagen Iα2) and kappa-elastin peptides (a fragment of elastin) [77]. Matricryptins can also induce monocyte activation and differentiation into macrophages [78].

**Fig. 1:**
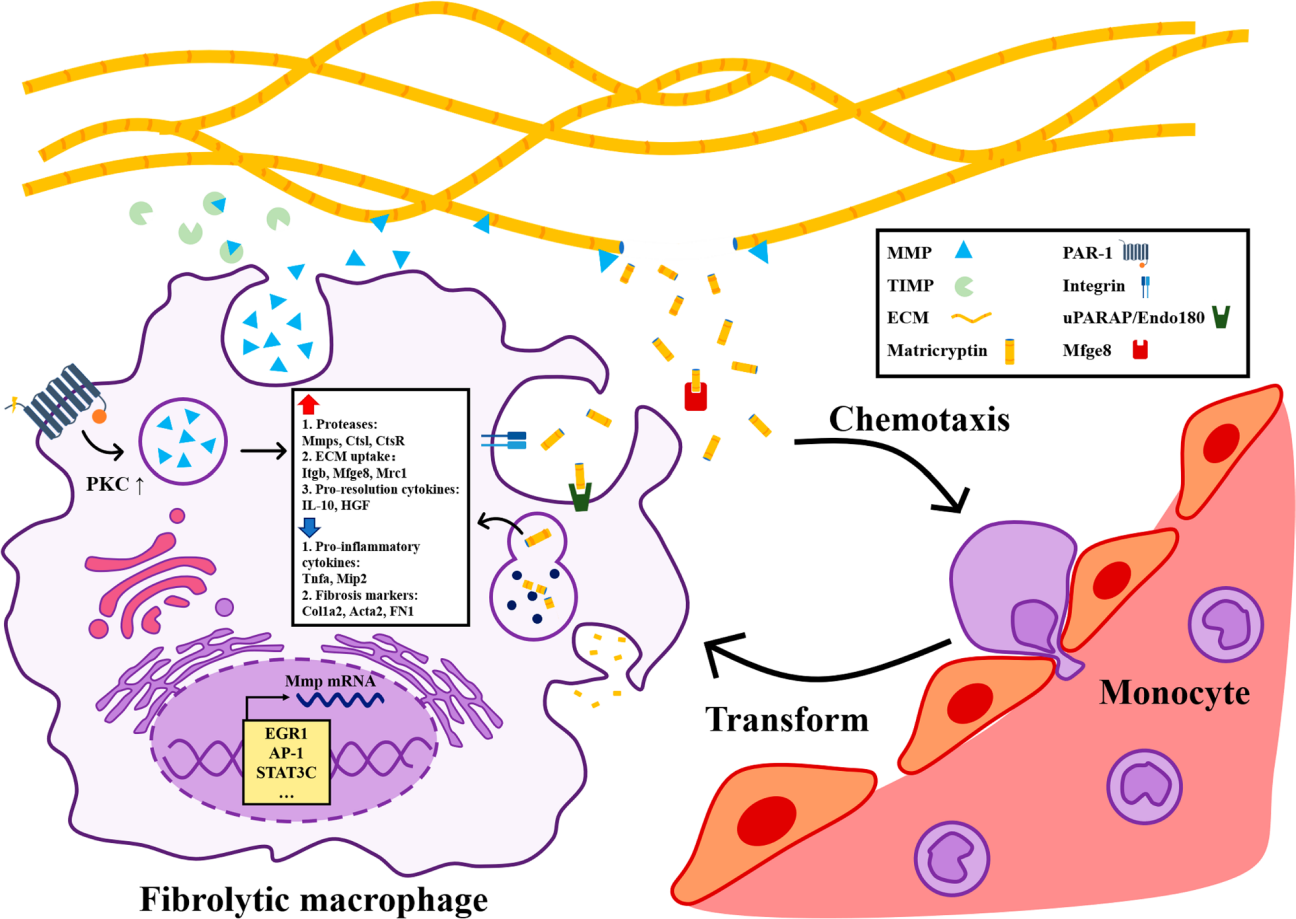
Macrophage-MMP-ECM interaction in fibrosis. Fibrolytic macrophages can degrade the ECM by secreting MMPs and phagocytizing ECM components in fibrotic sites. Regulated at the transcriptional and secretory levels, MMPs are released by fibrolytic macrophages to hydrolyze ECM components. Matricryptins, hydrolysis products of ECM degradation, are ingested by fibrolytic macrophages through the lysosomal pathway. Both MMPs and matricryptins can regulate the function of fibrolytic macrophages, enhancing their ability to resolve fibrosis. In addition, matricryptins exert a chemotactic effect on monocytes in peripheral blood, which may induce the transformation into fibrolytic macrophages due to inducing stimuli in the microenvironment

Macrophages are the main cells that secrete MMPs, and MMPs are important proteases in ECM degradation. Based on the relationships between fibrolytic macrophages, MMPs and ECM, macrophage-MMP-ECM interaction is established in fibrotic sites (Figure 1).

### The double-edged sword in fibrosis? Profibrotic effect of macrophage-MMP-ECM interaction

Although the macrophage-MMP-ECM interaction can promote ECM degradation and fibrosis regression, the ECM continues to deposit with the progression of fibrosis, making the situation worse and possibly irreversible. This outcome suggests that many factors in the fibrotic microenvironment hinder the occurrence and advancement of ECM degradation.

Macrophages can be anti-fibrotic, but the phenotype and function of macrophages vary markedly, and therefore, macrophages may become promoters of fibrosis. In the early stage of injury in organs such as lung, skin and liver, M1 macrophages promote ECM deposition and fibrosis by producing proinflammatory cytokines (IL-1, IL-6, IL-12, etc.) and activating fibroblasts and pericytes [34]. The phenotype and function of tissue-resident macrophages are determined by the microenvironment. Injury- and inflammation-related factors, including pathogen-associated molecular patterns (PAMPs), DAMPs and proinflammatory cytokines, induce macrophage polarization to the M1 subtype, [79] and M1 macrophages can be transformed into M2 macrophages (some of these cells are anti-profibrotic macrophages) through stimuli, such as cell debris, IL-1R and prostaglandin E2 (PGE2) [80]. Therefore, the dominance of profibrotic macrophages and the insufficient proportion and function of anti-fibrotic macrophages are possible reasons for the failure of fibrosis remission.

MMPs not only can degrade excessive ECM in fibrosis but can also disrupt the normal structure of the ECM, resulting in tissue damage and aggravated fibrosis. In renal fibrosis, TGF-β-activated macrophages release MMP-9, which specifically degrades the tubular basement membrane, leading to tubular cell EMT and ultimately kidney fibrosis. Inhibition of MMP-9 activity can reduce macrophage recruitment and infiltration, prevent the EMT and reduce the degree of fibrosis, making MMP-9 a potential therapeutic target in renal fibrosis[81]. Matricryptins produced by ECM degradation also contribute to inflammation and fibrosis. Moreover, MMP cleavage can enhance the degree to which chemokines, such as osteopontin, recruit macrophages, thereby promoting macrophage migration in the early stage of renal fibrosis [82]. In Ang II-induced skin and perivascular fibrosis, MMP-12 deficiency prevents the enrichment of M2 macrophages in fibrotic sites and attenuates the degree of fibrosis [83].

Because of these profibrotic factors, macrophage-MMP-ECM interaction may promote ECM deposition rather than degradation in fibrotic sites. In this regard, eliminating profibrotic factors or enhancing anti-fibrotic factors may be a therapeutic strategy for fibrosis because they can induce macrophage switching into a fibrolytic phenotype by altering upstream signals or inhibiting the profibrotic functions of certain MMPs.

## Conclusions

ECM deposition is not only a feature of fibrotic diseases but is also the core pathological process in fibrosis. Research on ECM degradation in fibrosis is increasing, and targeting ECM degradation to treat fibrosis shows great potential. On the one hand, this approach can significantly alleviate established fibrosis through symptomatic treatment. More importantly, the restoration of ECM homeostasis can improve the tissue microenvironment and potentially inhibit overactivation of fibroblasts. The macrophage-MMP-ECM interaction is a major pathway in ECM degradation during fibrosis, and macrophages are the main producers and regulators of MMPs. Hence, we suggest that precisely regulating the function of fibrolytic macrophages may better contribute to the degradation of ECM and the alleviation of fibrosis. Further investigations are needed to identify the role played by fibrolytic macrophages in fibrosis, including 1) the origin and transformation of fibrolytic macrophages in different stages of fibrotic disease and 2) the mechanism of interaction between fibrolytic macrophages and fibroblasts. Nonetheless, the macrophage-MMP-ECM interaction can lead to better understanding of the pathogenesis and progression of fibrosis from a different angle, and targeting this interaction may be a promising therapeutic strategy for fibrosis.

## Data Availability

Not applicable (the data in this review all came from the referenced articles).

## Abbreviations

TIMP: Tissue inhibitor of metalloproteinase
IPF: Idiopathic pulmonary fibrosis
AF: Atrial fibrillation
α-SMA: α-Smooth muscle actin
EMT: Epithelial-mesenchymal transition
UC: Ulcerative colitis
CD: Crohn’s disease
col (V): Collagen V
CCL4: Carbon tetrachloride
MT1-MMP: Membrane-type1 MMP
LRP1: Low-density lipoprotein receptor-related protein 1
FGF-2: Fibroblast growth factor-2
MFGE8: Milk fat globule epidermal growth factor 8
NOX2: NADPH oxidase 2
PPARγ: Peroxisome proliferator-activated receptor γ
HGF: Hepatic growth factor
MCP-3: Monocyte chemoattractant protein-3
uPARAP: Urokinase plasminogen activator receptor–associated protein
CSPG: Chondroitin sulfate proteoglycan
PAMP: Pathogen-associated molecular pattern
DAMP: Damage-associated molecular pattern
PGE-2: Prostaglandin E2
Ang: Angiopoietin
PAR-1: Protease-activated receptor 1

## References

[1] Vasse GF (2021). Macrophage-stroma interactions in fibrosis: biochemical, biophysical, and cellular perspectives. J Pathol.

[2] Wynn TA, Ramalingam TR (2012). Mechanisms of fibrosis: therapeutic translation for fibrotic disease. Nat Med.

[3] Conrad N (2018). Temporal trends and patterns in heart failure incidence: a population-based study of 4 million individuals. Lancet.

[4] Volk ML (2020). Burden of cirrhosis on patients and caregivers. Hepatol Commun.

[5] Kaplan GG (2015). The global burden of IBD: from 2015 to 2025. Nat Rev Gastroenterol Hepatol.

[6] Bonnans C, Chou J, Werb Z (2014). Remodelling the extracellular matrix in development and disease. Nat Rev Mol Cell Biol.

[7] Rabelink TJ (2017). Heparanase: roles in cell survival, extracellular matrix remodelling and the development of kidney disease. Nat Rev Nephrol.

[8] Duarte S, Baber J, Fujii T, Coito AJ (2015). Matrix metalloproteinases in liver injury, repair and fibrosis. Matrix Biol.

[9] Sun Z (2022). Circadian rhythm disorders elevate macrophages cytokines release and promote multiple tissues/organs dysfunction in mice. Physiol Behav.

[10] Caligiuri A, Gentilini A, Pastore M, Gitto S, Marra F (2021). Cellular and molecular mechanisms underlying liver fibrosis regression. Cells.

[11] Hynes RO, Naba A (2012). Overview of the matrisome–an inventory of extracellular matrix constituents and functions. Cold Spring Harb Perspect Biol.

[12] Theocharis AD, Skandalis SS, Gialeli C, Karamanos NK (2016). Extracellular matrix structure. Adv Drug Deliv Rev.

[13] Murphy-Ullrich JE, Sage EH (2014). Revisiting the matricellular concept. Matrix Biol.

[14] Rozario T, DeSimone DW (2010). The extracellular matrix in development and morphogenesis: a dynamic view. Dev Biol.

[15] Schuppan D, Ruehl M, Somasundaram R, Hahn EG (2001). Matrix as a modulator of hepatic fibrogenesis. Semin Liver Dis.

[16] Kimura G (2008). Progress in nephrology during this year: kidney and hypertension. Nihon Jinzo Gakkai Shi.

[17] Le Bousse-Kerdiles MC, Martyre MC, Samson M (2008). Cellular and molecular mechanisms underlying bone marrow and liver fibrosis: a review. Eur Cytokine Netw.

[18] Costa-Barney V, Delgado-Villarreal AF, Leguizamo AM, Vargas RD (2020). Diagnostic challenge between crohn’s disease and intestinal tuberculosis in chronic diarrhea with ulcerated jejunal stenosis: a case report. Rev Gastroenterol Peru.

[19] Henderson NC, Rieder F, Wynn TA (2020). Fibrosis: from mechanisms to medicines. Nature.

[20] Thannickal VJ, Toews GB, White ES, Lynch JP, Martinez FJ (2004). Mechanisms of pulmonary fibrosis. Annu Rev Med.

[21] Reese-Petersen AL, Olesen MS, Karsdal MA, Svendsen JH, Genovese F (2020). Atrial fibrillation and cardiac fibrosis: a review on the potential of extracellular matrix proteins as biomarkers. Matrix Biol.

[22] Barry-Hamilton V (2010). Allosteric inhibition of lysyl oxidase-like-2 impedes the development of a pathologic microenvironment. Nat Med.

[23] Deng Z (2020). The extracellular matrix and mechanotransduction in pulmonary fibrosis. Int J Biochem Cell Biol.

[24] Liu F (2015). Mechanosignaling through YAP and TAZ drives fibroblast activation and fibrosis. Am J Physiol Lung Cell Mol Physiol.

[25] Liu MN, Luo G, Gao WJ, Yang SJ, Zhou H (2021). miR-29 family: A potential therapeutic target for cardiovascular disease. Pharmacol Res.

[26] Giuffrida P, Biancheri P, MacDonald TT (2014). Proteases and small intestinal barrier function in health and disease. Curr Opin Gastroenterol.

[27] Frantz C, Stewart KM, Weaver VM (2010). The extracellular matrix at a glance. J Cell Sci.

[28] Mortensen JH (2015). Fragments of citrullinated and mmp-degraded vimentin and MMP-degraded type III collagen are novel serological biomarkers to differentiate crohn’s disease from ulcerative colitis. J Crohns Colitis.

[29] Bennett RG (2009). Relaxin and its role in the development and treatment of fibrosis. Transl Res.

[30] Wilkes DS (2015). Oral immunotherapy with type V collagen in idiopathic pulmonary fibrosis. Eur Respir J.

[31] Atabai K, Yang CD, Podolsky MJ (2020). You say you want a resolution (of fibrosis). Am J Respir Cell Mol Biol.

[32] Shapouri-Moghaddam A (2018). Macrophage plasticity, polarization, and function in health and disease. J Cell Physiol.

[33] Mills CD, Kincaid K, Alt JM, Heilman MJ, Hill AM (2000). M-1/M-2 macrophages and the Th1/Th2 paradigm. J Immunol.

[34] Smigiel KS, Parks WC (2018). Macrophages, wound healing, and fibrosis: recent insights. Curr Rheumatol Rep.

[35] Haider N (2019). Transition of macrophages to fibroblast-like cells in healing myocardial infarction. J Am Coll Cardiol.

[36] Wynn TA, Vannella KM (2016). Macrophages in tissue repair, regeneration, and fibrosis. Immunity.

[37] Kisseleva T, Brenner DA (2012). The phenotypic fate and functional role for bone marrow-derived stem cells in liver fibrosis. J Hepatol.

[38] Shaw OM, Hurst RD, Harper JL (2016). Boysenberry ingestion supports fibrolytic macrophages with the capacity to ameliorate chronic lung remodeling. Am J Physiol Lung Cell Mol Physiol.

[39] Nishi M (2020). Mesenchymal Stem cells induce a fibrolytic phenotype by regulating mmu-miR-6769b-5p expression in macrophages. Stem Cells Dev.

[40] Vizovisek M, Fonovic M, Turk B (2019). Cysteine cathepsins in extracellular matrix remodeling: extracellular matrix degradation and beyond. Matrix Biol.

[41] Atabai K (2009). Mfge8 diminishes the severity of tissue fibrosis in mice by binding and targeting collagen for uptake by macrophages. J Clin Invest.

[42] Rantakari P (2016). Stabilin-1 expression defines a subset of macrophages that mediate tissue homeostasis and prevent fibrosis in chronic liver injury. Proc Natl Acad Sci.

[43] Ramachandran P (2012). Differential Ly-6C expression identifies the recruited macrophage phenotype, which orchestrates the regression of murine liver fibrosis. Proc Natl Acad Sci.

[44] Butenko S (2020). Transcriptomic analysis of monocyte-derived non-phagocytic macrophages favors a role in limiting tissue repair and fibrosis. Front Immunol.

[45] Shah PK (1995). Human monocyte-derived macrophages induce collagen breakdown in fibrous caps of atherosclerotic plaques. potential role of matrix-degrading metalloproteinases and implications for plaque rupture. Circulation.

[46] Dancer RC, Wood AM, Thickett DR (2011). Metalloproteinases in idiopathic pulmonary fibrosis. Eur Respir J.

[47] Lech M, Anders HJ (1832). Macrophages and fibrosis: How resident and infiltrating mononuclear phagocytes orchestrate all phases of tissue injury and repair. Biochim Biophys Acta.

[48] Fallowfield JA (2007). Scar-associated macrophages are a major source of hepatic matrix metalloproteinase-13 and facilitate the resolution of murine hepatic fibrosis. J Immunol.

[49] Arora PD, Manolson MF, Downey GP, Sodek J, McCulloch CA (2000). A novel model system for characterization of phagosomal maturation, acidification, and intracellular collagen degradation in fibroblasts. J Biol Chem.

[50] Martinez-Pomares L (2006). Carbohydrate-independent recognition of collagens by the macrophage mannose receptor. Eur J Immunol.

[51] East L (2003). A targeted deletion in the endocytic receptor gene Endo180 results in a defect in collagen uptake. EMBO Rep.

[52] Ma PF (2017). Cytotherapy with M1-polarized macrophages ameliorates liver fibrosis by modulating immune microenvironment in mice. J Hepatol.

[53] Harty MW (2008). Hepatic macrophages promote the neutrophil-dependent resolution of fibrosis in repairing cholestatic rat livers. Surgery.

[54] Gharib SA (2014). MMP28 promotes macrophage polarization toward M2 cells and augments pulmonary fibrosis. J Leukoc Biol.

[55] Keane MP (1999). Neutralization of the CXC chemokine, macrophage inflammatory protein-2, attenuates bleomycin-induced pulmonary fibrosis. J Immunol.

[56] Balce DR (2011). Alternative activation of macrophages by IL-4 enhances the proteolytic capacity of their phagosomes through synergistic mechanisms. Blood.

[57] Yoon YS (2015). PPARgamma activation following apoptotic cell instillation promotes resolution of lung inflammation and fibrosis via regulation of efferocytosis and proresolving cytokines. Mucosal Immunol.

[58] Deniset JF (2019). Gata6(+) pericardial cavity macrophages relocate to the injured heart and prevent cardiac fibrosis. Immunity.

[59] Santos JC (2014). Endogenous cathelicidin production limits inflammation and protective immunity to Mycobacterium avium in mice. Immun Inflamm Dis.

[60] Rohani MG, Parks WC (2015). Matrix remodeling by MMPs during wound repair. Matrix Biol.

[61] Broder C (2013). Metalloproteases meprin alpha and meprin beta are C- and N-procollagen proteinases important for collagen assembly and tensile strength. Proc Natl Acad Sci U S A.

[62] Joshi CR, Stacy S, Sumien N, Ghorpade A, Borgmann K (2020). Astrocyte HIV-1 tat differentially modulates behavior and brain MMP/TIMP balance during short and prolonged induction in transgenic mice. Front Neurol.

[63] Neidhart B (2021). Tissue inhibitor of metalloproteinase (TIMP) peptidomimetic as an adjunctive therapy for infectious keratitis. Biomacromol.

[64] Laviades C (1998). Abnormalities of the extracellular degradation of collagen type I in essential hypertension. Circulation.

[65] Bjornstad JL (2008). Alterations in circulating activin A, GDF-15, TGF-beta3 and MMP-2, -3, and -9 during one year of left ventricular reverse remodelling in patients operated for severe aortic stenosis. Eur J Heart Fail.

[66] Pellicoro A (2012). Elastin accumulation is regulated at the level of degradation by macrophage metalloelastase (MMP-12) during experimental liver fibrosis. Hepatology.

[67] Leco KJ (2001). Spontaneous air space enlargement in the lungs of mice lacking tissue inhibitor of metalloproteinases-3 (TIMP-3). J Clin Invest.

[68] Newby AC (2016). Metalloproteinase production from macrophages—a perfect storm leading to atherosclerotic plaque rupture and myocardial infarction. Exp Physiol.

[69] Kessenbrock K, Plaks V, Werb Z (2010). Matrix metalloproteinases: regulators of the tumor microenvironment. Cell.

[70] Sternlicht MD, Werb Z (2001). How matrix metalloproteinases regulate cell behavior. Annu Rev Cell Dev Biol.

[71] Nighot M (2021). Matrix metalloproteinase MMP-12 promotes macrophage transmigration across intestinal epithelial tight junctions and increases severity of experimental colitis. J Crohns Colitis.

[72] McQuibban GA (2000). Inflammation dampened by gelatinase A cleavage of monocyte chemoattractant protein-3. Science.

[73] Lescoat A (2020). Combined anti-fibrotic and anti-inflammatory properties of JAK-inhibitors on macrophages in vitro and in vivo: Perspectives for scleroderma-associated interstitial lung disease. Biochem Pharmacol.

[74] McKleroy W, Lee TH, Atabai K (2013). Always cleave up your mess: targeting collagen degradation to treat tissue fibrosis. Am J Physiol Lung Cell Mol Physiol.

[75] Shechter R, Raposo C, London A, Sagi I, Schwartz M (2011). The glial scar-monocyte interplay: a pivotal resolution phase in spinal cord repair. PLoS ONE.

[76] Lindsey ML (2015). A novel collagen matricryptin reduces left ventricular dilation post-myocardial infarction by promoting scar formation and angiogenesis. J Am Coll Cardiol.

[77] de Castro Bras LE, Frangogiannis NG (2020). Extracellular matrix-derived peptides in tissue remodeling and fibrosis. Matrix Biol.

[78] Luikart SD (2009). Mactinin, a fragment of cytoskeletal alpha-actinin, is a novel inducer of heat shock protein (Hsp)-90 mediated monocyte activation. BMC Cell Biol.

[79] Tang PM, Nikolic-Paterson DJ, Lan HY (2019). Macrophages: versatile players in renal inflammation and fibrosis. Nat Rev Nephrol.

[80] Das A (2015). Monocyte and macrophage plasticity in tissue repair and regeneration. Am J Pathol.

[81] Zhao H (2013). Matrix metalloproteinases contribute to kidney fibrosis in chronic kidney diseases. World J Nephrol.

[82] Tan TK (2013). Matrix metalloproteinase-9 of tubular and macrophage origin contributes to the pathogenesis of renal fibrosis via macrophage recruitment through osteopontin cleavage. Lab Invest.

[83] Stawski L, Haines P, Fine A, Rudnicka L, Trojanowska M (2014). MMP-12 deficiency attenuates angiotensin II-induced vascular injury, M2 macrophage accumulation, and skin and heart fibrosis. PLoS ONE.

