# Correction: New insights into fibrosis from the ECM degradation perspective: the macrophage-MMP-ECM interaction

**DOI:** 10.1186/s13578-022-00881-9

**Published:** 2022-08-26

**Authors:** Xiangyu Zhao, Jiayin Chen, Hongxiang Sun, Yao Zhang, Duowu Zou

**Affiliations:** 1 Department of Gastroenterology, Ruijin Hospital, Shanghai Jiao Tong University School of Medicine, Shanghai, China; 2 Department of Immunology and Microbiology, Shanghai Institute of Immunology, Shanghai Jiao Tong University School of Medicine, Shanghai, China; 3 The State Key Laboratory of Oncogenes and Related Genes, Shanghai Jiao Tong University School of Medicine, Shanghai, China

## Correction: Cell & Bioscience (2022) 12:117 10.1186/s13578-022-00856-w

In the original version of this article [1], the corresponding authorship for the author Yao Zhang was missed and it has been updated with this correction.

The original article has been corrected.
